# Supplementary material for: Genetic association analysis of human median voice pitch identifies a common locus for tonal and non-tonal languages
Source: Commun Biol. 2024 May 7;7:540. doi: 10.1038/s42003-024-06198-2 (PMC11076565; doi:10.1038/s42003-024-06198-2)
Supplement: Supplementary file 2 — Supplementary Information [file 42003_2024_6198_MOESM2_ESM.pdf]

Supplementary for

Genetic association analysis of human median voice pitch identifies a common locus for tonal and non-tonal languages

The Supplementary contained the following contents:

- Supplementary Notes 1-3
- Supplementary Figures 1-5
- Supplementary Tables 1-7
- Supplementary Data 1. SNPs associated with pitch (median F0) in cross-population meta-analysis. (in a separate MS Excel file)

Supplementary Note 1: A Comparison Between Different F0 Quantiles

The Iceland study used the F0 median as the proxy of the pitch, as it is more resistant to outliers than the mean. Here we ask, is it representative of the pitch in Chinese women's spontaneous speech? We first compared the F0 median in our study with the 7,278 females in the Iceland study. When we compared the standard deviation and skewness of F0 (F0_SD) for each subject in our study with the Iceland females. Our samples had a significantly larger standard deviation and skewness. This means that there was a much more considerable variability and asymmetry in the pitch distribution for Chinese women's spontaneous speech.

We then look at other quantile values in F0 series for each subject. The distribution of the eleven quantile values (0th, 10th, 20, …, 100th) of F0 is in **Supplementary Figure 1**. The 0th quantile and the 100th quantile had a wider range compared to the middle quantiles. This could be due to

outliers brought by environmental noise and phonetic reliability. It may, otherwise, indicate differences in their vocal range between the subjects.

Next, we checked whether median F0 was the most frequent value in overall pitch values in speech. For each person and their given time series of F0 values, we calculated the frequency of these values in different quantile bands (0-1th quantile, 1-2nd quantiles, …, 99-100th quantile). Then we calculated the mean of these frequencies in cases and controls. A higher frequency means this F0 quantile accounted for more of the time in their speech. The results are shown in **Supplementary Figure 2**. The most frequent quantile values are around the 25th quantile (first quartile), both for cases and controls, indicating that the first quartile of F0 accounted for most of the time in their speech and may be more representative of the overall pitch in our study.

The next thing we checked was which quantile value of F0 was more reliable. Assuming that a biologically related phenotype should show high test-retest reliability, we split the audio for each person into two halves and calculated the quantile values of F0 separately. Then we calculated the correlation for the same F0 quantile in these two halves. A higher Pearson's r-value meant that the same F0 quantile measured from the first half of the audio was highly correlated with that measured in the second half of the audio, thus indicating high reliability. The results are shown in **Supplementary Figure 3**. Values around the 30th quantile showed the highest reliability, suggesting that they might be more biologically related.

We compared the heritability of the first/third quartile of F0 with the median F0. As shown in **Supplementary Table 3**, In the whole group (case+control), the heritability of the first quartile and median were close. However, in the control group, the heritability of the median is lower than the first quartile of F0.

To summarize, the Chinese female speakers spoke with F0 being more frequently around the first quartile of their F0 range, rather than the median. And the median F0 did not have the highest reliability. Thus, we thought the F0 median was not the most representative value among all the F0 quantiles in our study. The first quartile might be more reliable. It had a genome-wide significant association with rs11046212, which was stronger than the median F0. However, it is unknown what's the difference in the physiological process underlying the median F0 and the first quartile of F0. Besides, methods other than quartile values have not been considered, such as outlier removal or average methods.

Supplementary Note 2: Hospital Meta-analysis of two top SNPs

Since we observed variation between hospitals in the distribution of voice features, we also ran a hospital-level meta-analysis to confirm that the associations between the two top SNPs (rs11046212 and rs10859172) and pitch were not heterogeneous between hospitals. The sample distributions across hospitals are shown in **Supplementary Table 5**.

At stage 1, for each hospital, a multivariate linear regression model was fitted for pitch as the dependent variable using one SNP and covariates as the predictor variables. The covariates were the same as in the GWAS analysis, including MDD, age, 20 genetic PCs, as well as other variables listed in **Supplementary Table 2**. We applied a rank-based inverse normal transformation to the pitch. At stage 2, beta coefficients and standard errors from stage 1 were pooled using random-effects meta-analyses[4]. Small hospitals with too few samples (N<100) were grouped into one to get stable estimation.

The results of pooled associations with pitch were: rs11046212-T, $\beta = 0.08\,SD$, $P = 9.72 \times 10^{-5}$; and rs10859172-C, $\beta = -0.07\,SD$, $P = 8.92 \times 10^{-5}$). P-values for heterogeneity

tests of the associations were 0.62 for rs11046212-T and 0.44 for rs10859172-C, showing no evidence of heterogeneity.

Supplementary Note 3: Interview protocol

All subjects were interviewed using a computerized assessment system. Interviewers were postgraduate medical students, junior psychiatrists, or senior nurses, trained by the CONVERGE team for a minimum of 1 week. Interviews were recorded and the research team listened to at least two interviews from each interviewer to identify any errors in the way questions were asked and answers were interpreted.

The interview protocol acquired the following assessments for psychopathology: i) CIDI (WHO 1997) section of MDD expanded to include a "deep" assessment of the DSM-IV A criteria for MDD, symptoms of DSM-IV melancholia, Beck's cognitive triad (helplessness, hopefulness, and worthlessness), and irritability/anxiety; ii) CIDI section on dysthymia; iii) sections from interviews in the Virginia Adult Twin Study of Psychiatric and Substance Use Disorders (VATSPSUD)[1] for generalized anxiety disorder, panic, and five phobia subtypes (agoraphobia and social, situational, animal, and blood injury phobias), (iv) brief assessments of premenstrual syndrome and postnatal depression[2,3], and (v) assessments of smoking/nicotine dependence (Heatherton et al., 1991) (alcohol and substance abuse were virtually absent in this study, so it was not assessed).

Additionally, four key environmental exposures known to be strongly associated with the risk of MDD were assessed in cases and controls: i) child sexual abuse; ii) parent-child relationships; iii) social support; and iv) stressful live events. Neuroticism was assessed using the full 23-item Eysenck personality questionnaire N scale. Family history of MDD was individually assessed in

parents and full siblings using the Family History Research Diagnostic Criteria. In each case, measures used are those developed, field-tested, and validated in the VATSPSUD studies [3].

Supplementary Figures

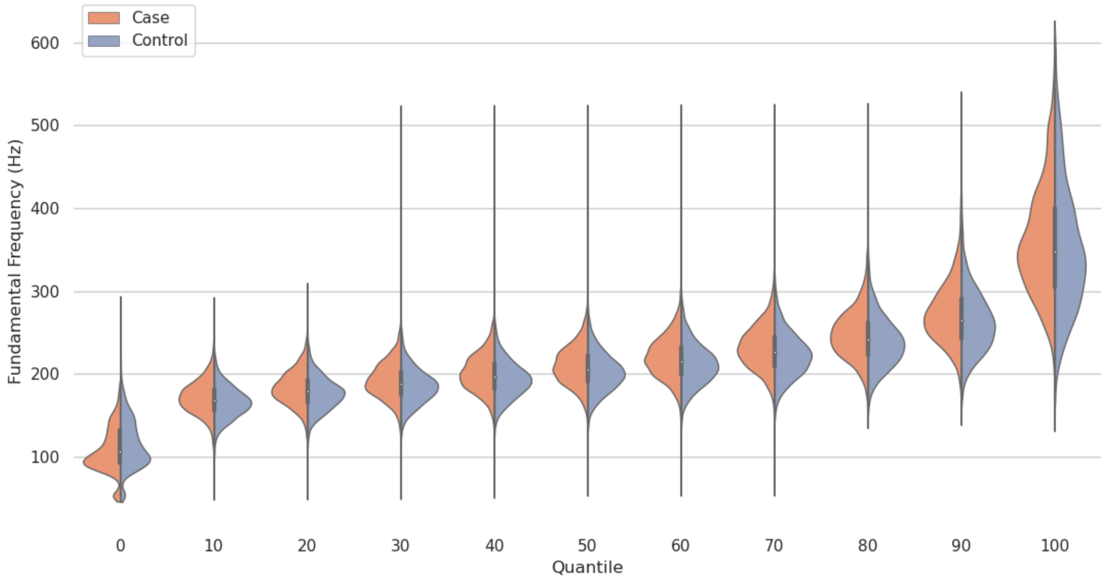

Supplementary Figure 1. Violin plot of the eleven F0 quantile values in major depressive disorder cases (left half) and controls (right half). For each subject, we calculated their eleven quantile values from the F0 series extracted from the audio segment. Then we plotted the distribution for each one of the 11 quantile values in cases and controls.

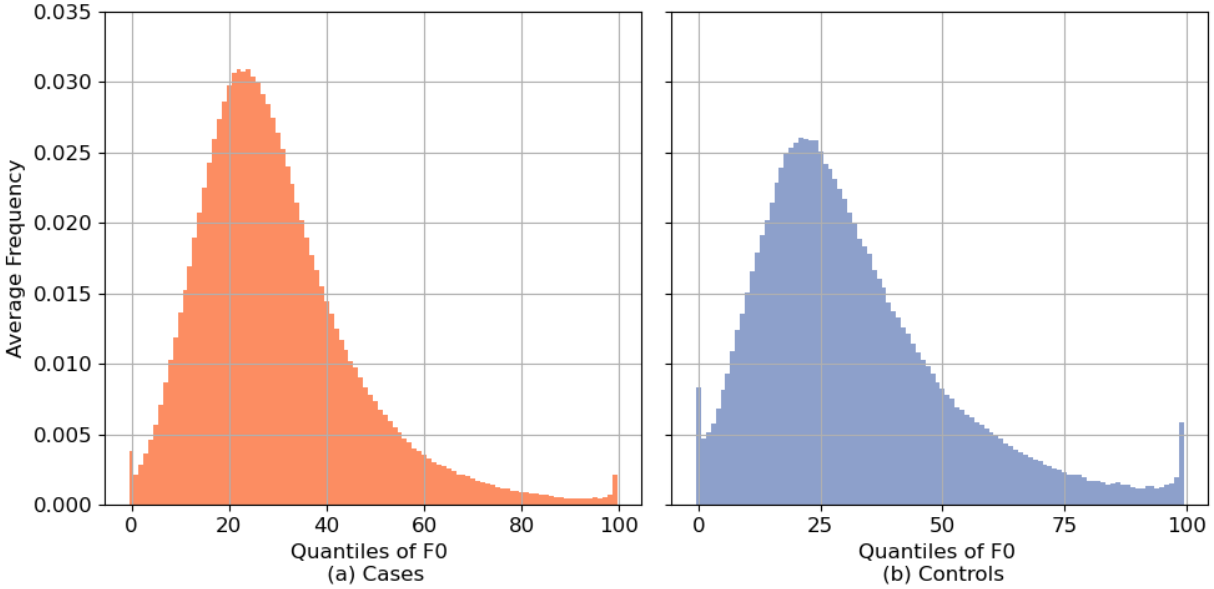

Supplementary Figure 2. Frequency of different F0 quantile values.

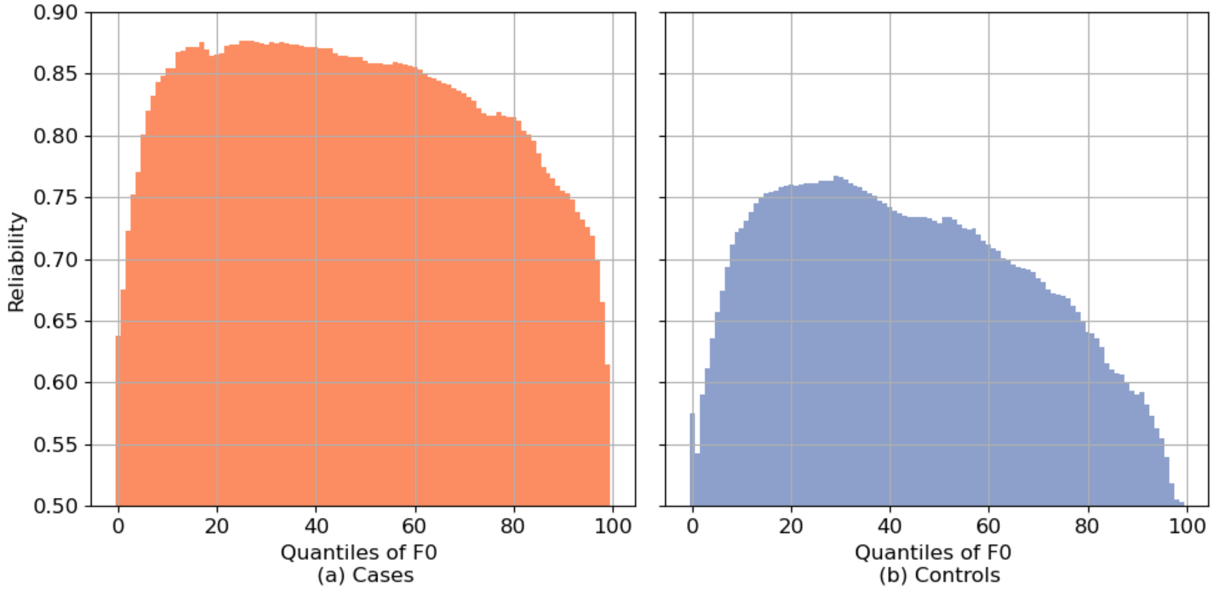

Supplementary Figure 3. Reliability of different F0 quantile values.

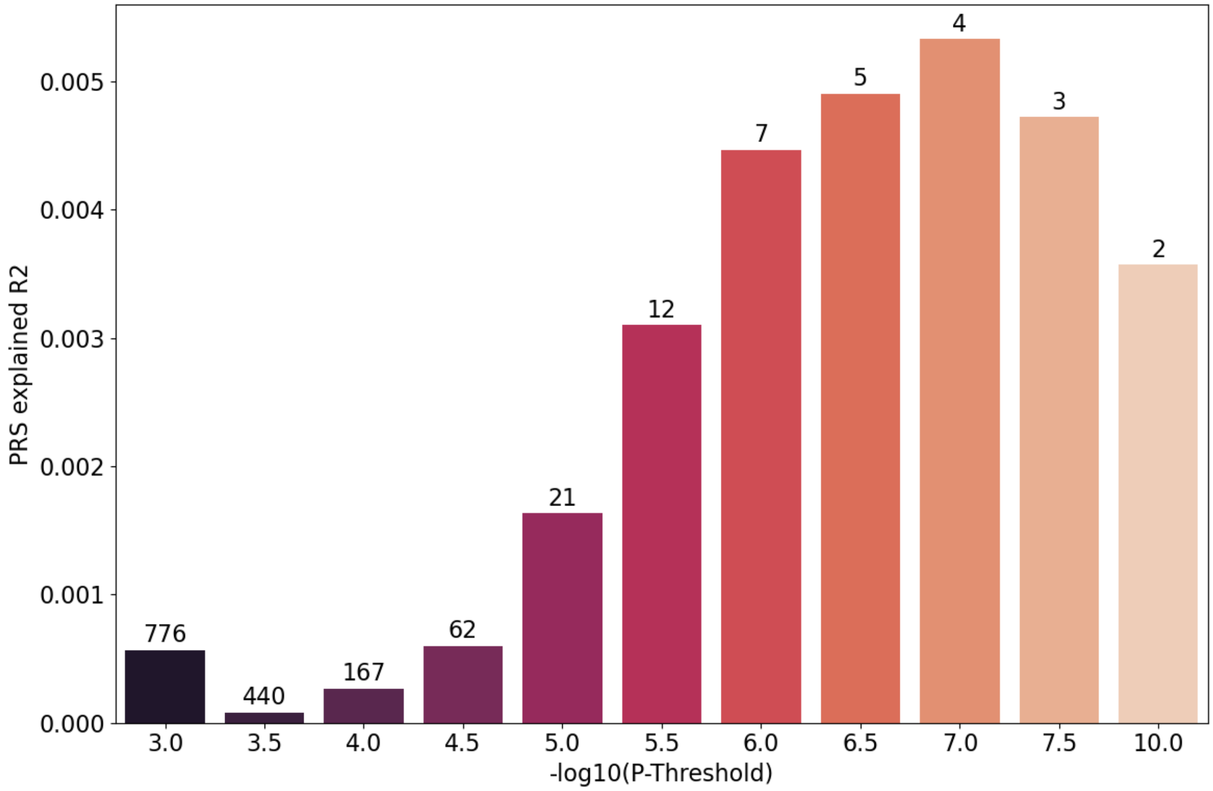

Supplementary Figure 4. The predictive performance of polygenic scores on MDD cases. The number of SNPs is labeled on each bar.

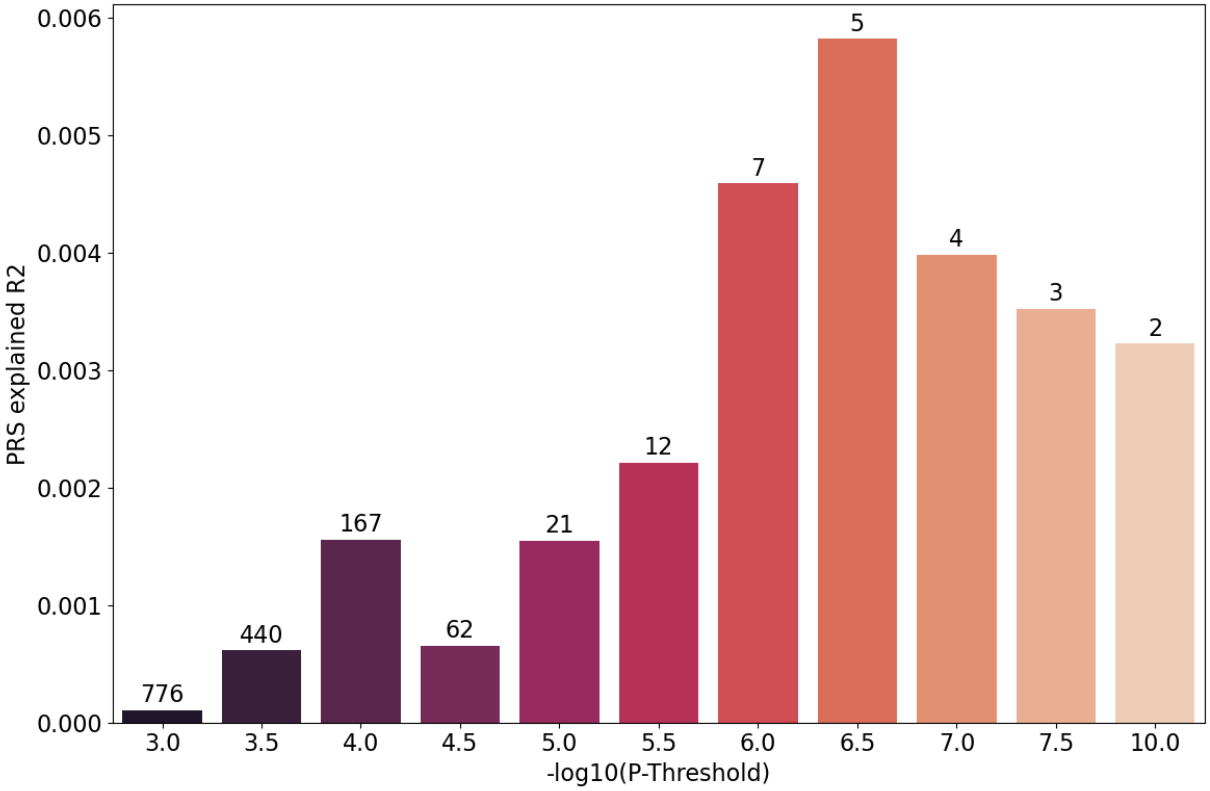

Supplementary Figure 5. The predictive performance of polygenic scores on controls. The number of SNPs is labeled on each bar.

Supplementary Tables

Supplementary Table 1. Sample Distribution.

		Cases	Controls
N		3641	4013
Interview Duration (min)	Mean	74.81	25.76
	SD	60.41	14.66
Segment Duration (s)	Mean	297.75	97.44
	SD	208.86	122.96
With an Accent		40.75%	47.46%
Nosie Level	1 (No Noise)	39.02%	46.13%
	2 (Low)	37.14%	32.86%
	3 (Mild)	18.38%	17.51%
	4 (High)	5.46%	3.50%
Age	Mean	44.02	47.55
	SD	8.92	5.53
% Education Level	No education	4.30%	3.19%
	Pre-school education	0.28%	0.28%
	Primary school	14.95%	11.58%
	Junior Middle school	27.89%	32.47%
	Senior Middle school	16.16%	22.47%
	Technical and vocational school	9.42%	9.24%
	Adult/radio/television schooling/evening education	0.66%	0.40%
	Junior College	12.42%	11.42%
	Bachelor Degree	11.51%	7.95%
	Master Degree	2.29%	0.73%
	Ph.D	0.14%	0.28%
% Occupation	Working now for pay	33.36%	52.50%
	Only temporarily laid off or sick leave	11.64%	3.55%
	Looking for work, unemployed	1.54%	0.77%
	Retired from a paid job	19.43%	18.99%
	Permanently disabled	0.06%	0.00%
	Keeping house/staying at home	28.52%	16.77%
	Going to school	0.06%	0.04%
	Other	5.39%	7.38%
% Social Class	Executives, business owners, major and lesser professionals	12.92%	12.54%
	Administrative Personnel, Minor Professionals, Clerical and Sales Worker	34.71%	25.52%
	Skilled Manual Employees	14.52%	13.95%
	Semi-Skilled and Unskilled Workers	23.69%	30.04%
	Other	14.16%	17.94%
% Marital Status	Married	83.04%	93.06%
	Separated	2.01%	0.62%
	Divorced	8.51%	3.02%
	Widowed	2.92%	2.72%
	Never married	3.52%	0.57%

Supplementary Table 2. Variables associated with pitch.

Variables	Level	Beta	SE	P	P_Bonferroni
accent		0.10	0.02	1.60E-05	**9.30E-04**
height		-0.07	0.01	5.84E-09	**3.39E-07**
BMI		-0.07	0.01	7.17E-08	**4.16E-06**
audio durations		0.04	0.01	1.88E-03	0.11
number_of_segements		0.04	0.01	2.91E-03	0.17
education	No education	0.23	0.06	4.55E-04	**0.03**
	Pre-school education	0.24	0.23	0.30	1.00
	Primary school	0.01	0.04	0.75	1.00
	Junior Middle school	-0.07	0.03	6.84E-03	0.40
	Senior Middle school	0.00	0.03	0.99	1.00
	Technical and vocational school	-0.11	0.04	0.01	0.53
	Adult/radio/television schooling/evening education	0.14	0.17	0.41	1.00
	Junior College	0.03	0.04	0.41	1.00
	Bachelor Degree	0.06	0.04	0.13	1.00
	Master Degree	0.29	0.10	2.62E-03	0.15
	Ph.D	0.25	0.28	0.37	1.00
marital status	Married	0.01	0.03	0.83	1.00
	Separated	-0.12	0.10	0.22	1.00
	Divorced	-0.01	0.05	0.84	1.00
	Widowed	-0.08	0.07	0.25	1.00
	Never married	0.18	0.08	0.03	1.00
noise level	1 (No Noise)	-0.16	0.02	4.98E-12	**2.89E-10**
	2 (Low)	0.00	0.02	0.87	1.00
	3 (Mild)	0.26	0.03	3.30E-19	**1.91E-17**
	4 (High)	0.19	0.06	5.72E-04	**0.03**
occupation	Working now for pay	0.03	0.02	0.29	1.00
	Only temporarily laid off or sick leave	0.03	0.05	0.45	1.00
	Looking for work, unemployed	0.03	0.11	0.78	1.00
	Retired from a paid job	-0.06	0.04	0.11	1.00
	Permanently disabled	-0.05	0.96	0.96	1.00
	Keeping house/staying at home	0.00	0.03	0.86	1.00
	Going to school	-0.73	0.55	0.19	1.00
	Other	-0.06	0.05	0.24	1.00
social class	Executives, business owners, major and lesser professionals	-0.01	0.04	0.71	1.00
	Administrative Personnel, Minor Professionals, Clerical and Sales Worker	0.00	0.03	0.97	1.00
	Skilled Manual Employees	-0.05	0.03	0.18	1.00
	Semi-Skilled and Unskilled Workers	0.08	0.03	4.43E-03	0.26
	Other	-0.07	0.03	0.05	1.00
genetic PCs	1	9.42	1.15	2.79E-16	**1.62E-14**
	2	-0.06	1.13	0.96	1.00
	3	0.10	1.12	0.93	1.00
	4	-0.41	1.14	0.72	1.00
	5	7.13	1.12	2.38E-10	**1.38E-08**
	6	-3.32	1.13	3.34E-03	0.19
	7	2.81	1.14	0.01	0.78
	8	-0.56	1.14	0.62	1.00
	9	0.78	1.13	0.49	1.00
	10	-0.30	1.13	0.79	1.00
	11	4.19	1.13	1.98E-04	**0.01**
	12	-0.02	1.13	0.99	1.00
	13	0.37	1.13	0.75	1.00
	14	1.30	1.16	0.26	1.00
	15	-1.70	1.14	0.14	1.00
	16	-1.13	1.15	0.33	1.00
	17	-0.17	1.14	0.88	1.00
	18	-0.23	1.16	0.84	1.00
	19	-1.06	1.14	0.35	1.00
	20	-0.24	1.18	0.84	1.00

Supplementary Table 3. SNP-based heritability of different quartiles of F0.

phenotype	group	Heritability	95%CI	
quartile1	case+control	0.18	0.09	0.28
	case	0.23	0.03	0.44
	control	0.20	0.02	0.39
quartile2	case+control	0.20	0.10	0.29
	case	0.21	0.00	0.41
	control	0.14	-0.05	0.33
quartile3	case+control	0.16	0.07	0.26
	case	0.17	-0.04	0.37
	control	0.07	-0.12	0.26

Supplementary Table 4. Associations between top SNPs and different quartiles of F0.

SNP	Quartile of F0	Beta	SD	P
rs11046212-T	First	0.096	0.018	3.32E-08
	Second (Median F0)	0.089	0.018	2.33E-07
	Third	0.079	0.018	4.05E-06
rs10859172-C	First	-0.064	0.016	1.71E-05
	Second (Median F0)	-0.078	0.016	2.06E-07
	Third	-0.086	0.016	2.53E-08

Supplementary Table 5. Sample distributions across hospitals.

Hospital	city	province	N	% case	% accent	% Noise L1	% Noise L2	% Noise L3	% Noise L4
1	Shanghai	Shanghai	835	99.88%	62.40%	35.21%	38.32%	18.80%	2.75%
2	Zhenjiang	Jiangsu	453	17.88%	49.45%	47.68%	26.27%	13.69%	3.53%
3	Hangzhou	Zhejiang	346	34.97%	39.02%	32.95%	27.75%	29.48%	8.67%
4	Xi'an	Shaanxi	344	33.72%	40.12%	65.70%	16.57%	9.30%	1.16%
5	Beijing	Beijing	335	35.22%	13.73%	25.67%	37.01%	25.97%	7.46%
6	Shenyang	Liaoning	310	23.87%	34.19%	50.65%	29.35%	8.39%	2.90%
7	Xi'an	Shaanxi	300	41.00%	35.00%	46.33%	30.67%	10.33%	3.00%
8	Hangzhou	Zhejiang	283	33.92%	30.04%	29.33%	36.04%	18.73%	2.47%
9	Taiyuan	Shaanxi	270	42.22%	25.93%	44.07%	35.56%	9.26%	1.48%
10	Shenyang	Liaoning	244	52.46%	18.44%	41.80%	40.57%	9.43%	7.38%
11	Jinan	Shandong	224	46.88%	41.52%	51.34%	21.88%	11.61%	7.14%
12	Shanghai	Shanghai	211	23.22%	65.40%	9.95%	37.91%	44.55%	7.11%
13	Nanchang	Jiangxi	211	47.87%	45.97%	25.59%	42.18%	23.22%	9.00%
14	Guangzhou	Guangdong	202	70.30%	33.17%	18.32%	53.96%	25.74%	0.99%
15	Nanjing	Jiangsu	188	31.38%	26.06%	55.32%	26.06%	10.64%	2.66%
16	Shanghai	Shanghai	177	14.12%	24.86%	64.97%	21.47%	11.86%	1.69%
17	Chengdu	Sichuan	172	29.65%	65.70%	41.86%	38.37%	16.86%	1.74%
18	Tianjin	Tianjin	148	56.76%	12.16%	62.84%	16.89%	10.81%	2.03%
19	Zhengzhou	Henan	146	54.11%	52.74%	32.19%	41.78%	16.44%	4.11%
20	Xinxiang	Henan	145	40.69%	66.90%	42.76%	30.34%	15.17%	6.90%
21	Baoding	Hebei	144	45.83%	45.14%	50.00%	20.83%	4.17%	2.78%
22	Guangzhou	Guangdong	134	34.33%	50.00%	31.34%	50.75%	14.18%	0.75%
23	Shenzhen	Guangdong	129	28.68%	27.13%	19.38%	50.39%	10.08%	0.00%
24	Siping	Jilin	119	85.71%	22.69%	48.74%	28.57%	9.24%	5.04%
25	Dalian	Liaoning	118	51.69%	46.61%	22.88%	33.05%	22.03%	14.41%
26	Haerbin	Heilongjiang	113	50.44%	13.27%	40.71%	21.24%	20.35%	6.19%
27	Lanzhou	Gansu	110	40.00%	27.27%	28.18%	47.27%	6.36%	9.09%
28	Beian	Heilongjiang	105	50.48%	1.90%	50.48%	36.19%	5.71%	0.00%
29	Wuhan	Hubei	98	15.31%	73.47%	32.65%	24.49%	35.71%	5.10%
30	Liaocheng	Shandong	95	55.79%	73.68%	18.95%	26.32%	37.89%	0.00%
31	Chongqing	Chongqing	94	44.68%	79.79%	24.47%	32.98%	41.49%	1.06%
32	Mianyang	Sichuan	88	47.73%	72.73%	39.77%	42.05%	2.27%	0.00%
33	Chongqing	Chongqing	86	75.58%	75.58%	13.95%	51.16%	19.77%	15.12%
34	Xi'an	Shaanxi	82	29.27%	23.17%	31.71%	56.10%	2.44%	0.00%
35	Guangzhou	Guangdong	82	39.02%	40.24%	24.39%	25.61%	46.34%	1.22%
36	Fuzhou	Fujian	76	53.95%	26.32%	18.42%	72.37%	9.21%	0.00%
37	Jining	Shandong	46	100.00%	17.39%	23.91%	60.87%	4.35%	2.17%
38	Ningbo	Zhejiang	43	55.81%	58.14%	62.79%	20.93%	2.33%	0.00%
39	Haerbin	Heilongjiang	38	81.58%	2.63%	13.16%	2.63%	39.47%	44.74%
40	Qingdao	Shandong	38	39.47%	34.21%	42.11%	26.32%	10.53%	0.00%
41	Changchun	Jilin	34	100.00%	44.12%	55.88%	23.53%	5.88%	0.00%
42	Changsha	Hunan	32	59.38%	40.63%	62.50%	12.50%	9.38%	6.25%
43	Huaian	Jiangsu	30	76.67%	43.33%	76.67%	16.67%	3.33%	0.00%
44	Suzhou	Jiangsu	29	48.28%	58.62%	24.14%	48.28%	20.69%	0.00%
45	Daqing	Heilongjiang	28	53.57%	39.29%	60.71%	7.14%	0.00%	0.00%
46	Huzhou	Zhejiang	26	65.38%	34.62%	69.23%	23.08%	0.00%	3.85%
47	Tangshan	Hebei	22	50.00%	18.18%	50.00%	45.45%	4.55%	0.00%
48	Hefei	Anhui	21	100.00%	66.67%	19.05%	33.33%	28.57%	0.00%
49	Mudanjiang	Heilongjiang	16	68.75%	0.00%	75.00%	6.25%	0.00%	0.00%
50	Shijiazhuang	Hebei	11	81.82%	54.55%	27.27%	63.64%	9.09%	0.00%
51	Beijing	Beijing	8	0.00%	0.00%	75.00%	25.00%	0.00%	0.00%
52	Weihai	Shandong	5	80.00%	40.00%	80.00%	0.00%	0.00%	20.00%
53	Anshan	Liaoning	4	100.00%	50.00%	25.00%	75.00%	0.00%	0.00%
54	Shantou	Guangdong	3	33.33%	66.67%	0.00%	0.00%	33.33%	66.67%
55	Haikou	Hainan	3	100.00%	33.33%	100.00%	0.00%	0.00%	0.00%

Supplementary Table 6. F0 statistics in Chinese and Icelandic. MDD: major depressive disorders. Pitch statistics of Iceland study is downloaded from artile supplementary: https://www.science.org/doi/suppl/10.1126/sciadv.abq2969/suppl_file/sciadv.abq2969_tables_s1_to_s11.zip

**Comparison between Iceland and Chinese females**						
F0 statistics	Chinese (N=7654)		Iceland (N=7278)		t-statistic	p-value
	Mean	SD	Mean	SD		
Median	206.25	26.31	203.01	21.16	8.31	1.06E-16
Standard Deviation	44.09	13.79	31.75	13.07	56.17	<1.23E-308
Skewness	1.34	0.98	0.72	1.28	32.97	3.14E-230
**Comparison between Chinese females MDD cases and controls***						
F0 statistics	Cases (N=3641)		Controls (N=4013)		t-statistic	p-value
	Mean	SD	Mean	SD		
Median	210.90	24.49	202.03	27.18	15.02	2.66E-50
Standard Deviation	44.68	12.27	43.56	15.02	3.56	3.71E-04
Skewness	1.34	0.91	1.33	1.05	0.70	0.49

Supplemental Table 7. The 95% credible set from the cross-ancestry fine-mapping analysis. SNP: SNP in the credible set that had the largest posterior inclusion probability (PIP). BP: The base pair coordinate of the SNP. REF_ALLELE: The reference allele of the SNP in each population (Icelandic, Chinese), separated by comma. ALT_ALLELE: The alternative allele of the SNP in each population, separated by comma. REF_FRQ: Frequency of the reference allele in each reference panel, separated by comma. BETA: Marginal per-allele effect size of the SNP with respect to the reference allele in each population, separated by comma.SE: The standard error of the marginal per-allele effect size of the SNP in each population, separated by comma. -LOG10P: -log10 of the marginal p-value of the SNP in each population, separated by comma. CS_PIP: Posterior inclusion probability (PIP) of the SNP. OVRL_PIP: Posterior inclusion probability (PIP) of the SNP in any of the credible set.

SNP	BP	REF_ALLELE	ALT_ALLELE	REF_FRQ	BETA	SE	-LOG10P	CS_PIP	OVRL_PIP
rs11046212	22006116	C,C	T,T	0.6143,0.7391	-0.111624,-0.0904709	0.0127896,0.0184057	17.585,6.05251	0.350885	0.350885
rs11046215	22010432	G,G	A,A	0.6143,0.7391	-0.111516,-0.0900623	0.0127896,0.0184057	17.5528,6.00339	0.296506	0.296506
rs10841900	22010665	G,G	A,A	0.6143,0.7391	-0.111516,-0.0900623	0.0127896,0.0184057	17.5528,6.00339	0.296506	0.296506
rs704185	22006369	C,C	G,G	0.5885,0.7411	-0.106039,-0.0908988	0.0126507,0.0184517	16.284,6.07678	0.0240154	0.0240154

References

1. Kendler, K. S. & Prescott, C. A. *Genes, Environment, and Psychopathology: Understanding the Causes of Psychiatric and Substance Use Disorders*. (Guilford Press, 2007).
2. Cox, J. L., Holden, J. M. & Sagovsky, R. Detection of postnatal depression: development of the 10-item Edinburgh Postnatal Depression Scale. *The British journal of psychiatry* **150**, 782–786 (1987).
3. Kendler, K. *et al.* Genetic and environmental factors in the aetiology of menstrual, premenstrual and neurotic symptoms: a population-based twin study. *Psychological Medicine* **22**, 85–100 (1992).
4. Viechtbauer, W. Conducting Meta-Analyses in R with the metafor Package. *Journal of Statistical Software* **36**, 1–48 (2010).
