# Supplementary material for: Genetic association analysis of human median voice pitch identifies a common locus for tonal and non-tonal languages
Source: Commun Biol. 2024 May 7;7:540. doi: 10.1038/s42003-024-06198-2 (PMC11076565; doi:10.1038/s42003-024-06198-2)
Supplement: Supplementary file 3 — Description of Additional Supplementary Data [file 42003_2024_6198_MOESM3_ESM.pdf]

Description of Additional Supplementary Files

File name: SupplementaryData1.xlsx

Description: SNPs associated with pitch (median F0) in cross-population meta- analysis.
