# Supplementary material for: Genetic association analysis of human median voice pitch identifies a common locus for tonal and non-tonal languages
Source: Commun Biol. 2024 May 7;7:540. doi: 10.1038/s42003-024-06198-2 (PMC11076565; doi:10.1038/s42003-024-06198-2)
Supplement: Supplementary file 5 — Reporting Summary [file 42003_2024_6198_MOESM5_ESM.pdf]

nature portfolio

Corresponding author(s): Jonathan Flint

Last updated by author(s): Feb 1, 2024

Reporting Summary

Nature Portfolio wishes to improve the reproducibility of the work that we publish. This form provides structure for consistency and transparency in reporting. For further information on Nature Portfolio policies, see our Editorial Policies and the Editorial Policy Checklist.

Statistics

For all statistical analyses, confirm that the following items are present in the figure legend, table legend, main text, or Methods section.

n/a	Confirmed	
☐	☒	The exact sample size (*n*) for each experimental group/condition, given as a discrete number and unit of measurement
☐	☒	A statement on whether measurements were taken from distinct samples or whether the same sample was measured repeatedly
☐	☒	The statistical test(s) used AND whether they are one- or two-sided<br>*Only common tests should be described solely by name; describe more complex techniques in the Methods section.*
☐	☒	A description of all covariates tested
☐	☒	A description of any assumptions or corrections, such as tests of normality and adjustment for multiple comparisons
☐	☒	A full description of the statistical parameters including central tendency (e.g. means) or other basic estimates (e.g. regression coefficient) AND variation (e.g. standard deviation) or associated estimates of uncertainty (e.g. confidence intervals)
☐	☒	For null hypothesis testing, the test statistic (e.g. *F*, *t*, *r*) with confidence intervals, effect sizes, degrees of freedom and *P* value noted<br>*Give P values as exact values whenever suitable.*
☒	☐	For Bayesian analysis, information on the choice of priors and Markov chain Monte Carlo settings
☐	☒	For hierarchical and complex designs, identification of the appropriate level for tests and full reporting of outcomes
☐	☒	Estimates of effect sizes (e.g. Cohen's *d*, Pearson's *r*), indicating how they were calculated

Our web collection on statistics for biologists contains articles on many of the points above.

Software and code

Policy information about availability of computer code

Data collection	The data is from the CONVERGE study, which has been previously reported (10.1038/nature14659). The voice data was collected during an interview through a computerized assessment system, as previously reported.
Data analysis	The median pitch was calculated using using the openSMILE package v2.4.2. Heritability estimation and GWAS was performed using LDAK v5.2. The genetic correlation of pitch between cases and controls were calculated using GCTA v.1.94.1 Linux. The meta-GWAS were implemented in Metal. Cross-ancestry fine-mapping analysis was performed in SuSiEx.

For manuscripts utilizing custom algorithms or software that are central to the research but not yet described in published literature, software must be made available to editors and reviewers. We strongly encourage code deposition in a community repository (e.g. GitHub). See the Nature Portfolio guidelines for submitting code & software for further information.

Data

Policy information about availability of data

All manuscripts must include a data availability statement. This statement should provide the following information, where applicable:

- Accession codes, unique identifiers, or web links for publicly available datasets
- A description of any restrictions on data availability
- For clinical datasets or third party data, please ensure that the statement adheres to our policy

The GWAS summary statistics of the median pitch in Chinese and the meta-GWAS are publicly available at FigShare (10.6084/m9.figshare.24995963). The GWAS summary data for the median pitch in the Iceland study are available at Zenodo (www.doi.org/10.5281/zenodo.7152461).

Research involving human participants, their data, or biological material

Policy information about studies with human participants or human data. See also policy information about sex, gender (identity/presentation), and sexual orientation and race, ethnicity and racism.

Reporting on sex and gender	Only biological female samples were included in this study to increase homogeneity. Sex is validated by their genetic data.
Reporting on race, ethnicity, or other socially relevant groupings	To increase homogeneity, all subjects were Han Chinese women with four Han Chinese grandparents.
Population characteristics	All subjects were Han Chinese women with four Han Chinese grandparents. Cases were excluded if they had a pre-existing history of bipolar disorder, psychosis or mental retardation. Cases were aged between 30 and 60 and had two or more episodes of MDD meeting DSM-IV criteria with the first episode occurring between 14 and 50 years of age, and had not abused drugs or alcohol before their first depressive episode.
Recruitment	CONVERGE collected cases of recurrent major depression from 58 provincial mental health centres and psychiatric departments of general medical hospitals in 45 cities and 23 provinces of China. Controls were recruited from patients undergoing minor surgical procedures at general hospitals (37%) or from local community centres (63%).
Ethics oversight	The study protocol was approved centrally by the Ethical Review Board of Oxford University (Oxford Tropical Research Ethics Committee) and the ethics committees of all participating hospitals in China. All interviewers were mental health professionals who are well able to judge decisional capacity. The study posed minimal risk (an interview and saliva sample). All participants provided their written informed consent.

Note that full information on the approval of the study protocol must also be provided in the manuscript.

Field-specific reporting

Please select the one below that is the best fit for your research. If you are not sure, read the appropriate sections before making your selection.

☒ Life sciences ☐ Behavioural & social sciences ☐ Ecological, evolutionary & environmental sciences

For a reference copy of the document with all sections, see nature.com/documents/nr-reporting-summary-flat.pdf

Life sciences study design

All studies must disclose on these points even when the disclosure is negative.

Sample size	In the CONVERGE study, a sample size of 6,000 cases and 6,000 controls was chosen on the basis of evidence available when the study was designed (in 2007) of the likely existence of genetic loci associated with major depressive disorder with odds ratio of 1.2 and above. The current study used all samples with available voice data from the CONVERGE study. Of the initial subjects recruited in CONVERGE, 8,322 subjects have interview recordings. To obtain the subjects' utterances, a group of undergraduates listened to the recordings to identify any voice segments from the subjects with a duration >2 seconds. Audio samples were excluded where the noise was so prominent that the content of the subject's speech could not be understood, which yielded 7,654 subjects with available segments.
Data exclusions	All recordings were manually listened to, and segments that contained only the patient's voice at an adequate quality for analyses were identified. Subjects without available voice data, or with too much noise in the voice data were excluded from analysis, which yields to a total sample size of 7,654.
Replication	We tested the reliability of the calculated median pitch, which are described in details in the Supplementary. Our GWAS results successfully replicated a genetic locus associated with pitch found in a previous GWAS study in Iceland. We've discussed the consistency of the GWAS results in our Chinese study with the Icelandic in the main text.
Randomization	This is nor an experimental study. Randomization is not applicable. In the GWAS, we adjusted for a group of covariates including age, height,

Randomization	BMI, education level, marital status, occupation, social class, accent, noise level, total audio durations, the total number of audio segments concatenated, and 20 genetic principal components (PCs).
Blinding	This is not an experimental study. Blinding is not applicable.

Reporting for specific materials, systems and methods

We require information from authors about some types of materials, experimental systems and methods used in many studies. Here, indicate whether each material, system or method listed is relevant to your study. If you are not sure if a list item applies to your research, read the appropriate section before selecting a response.

Materials & experimental systems

n/a	Involved in the study
☒	☐ Antibodies
☒	☐ Eukaryotic cell lines
☒	☐ Palaeontology and archaeology
☒	☐ Animals and other organisms
☒	☐ Clinical data
☒	☐ Dual use research of concern
☒	☐ Plants

Methods

n/a	Involved in the study
☒	☐ ChIP-seq
☒	☐ Flow cytometry
☒	☐ MRI-based neuroimaging

Plants

Seed stocks	*Report on the source of all seed stocks or other plant material used. If applicable, state the seed stock centre and catalogue number. If plant specimens were collected from the field, describe the collection location, date and sampling procedures.*
Novel plant genotypes	*Describe the methods by which all novel plant genotypes were produced. This includes those generated by transgenic approaches, gene editing, chemical/radiation-based mutagenesis and hybridization. For transgenic lines, describe the transformation method, the number of independent lines analyzed and the generation upon which experiments were performed. For gene-edited lines, describe the editor used, the endogenous sequence targeted for editing, the targeting guide RNA sequence (if applicable) and how the editor was applied.*
Authentication	*Describe any authentication procedures for each seed stock used or novel genotype generated. Describe any experiments used to assess the effect of a mutation and, where applicable, how potential secondary effects (e.g. second site T-DNA insertions, mosiacism, off-target gene editing) were examined.*
